# MicroRNA responses associated with *Salmonella enterica* serovar typhimurium challenge in peripheral blood: effects of miR-146a and IFN-γ in regulation of fecal bacteria shedding counts in pig

**DOI:** 10.1186/s12917-019-1951-4

**Published:** 2019-06-11

**Authors:** Tinghua Huang, Xiali Huang, Wang Chen, Jun Yin, Bomei Shi, Fangfang Wang, Wenzhao Feng, Min Yao

**Affiliations:** College of Animal Science, Yangtze University, Jingzhou, 434025 Hubei China

**Keywords:** *Salmonella*, Swine, microRNA, Immune response, miR-146a, IFN-γ

## Abstract

**Background:**

MicroRNAs are involved in a broad range of biological processes and are known to be differentially expressed in response to bacterial pathogens.

**Results:**

The present study identified microRNA responses in porcine peripheral blood after inoculation with the human foodborne pathogen *Salmonella enterica* serovar Typhimurium strain LT2. We compared the microRNA transcriptomes of the whole blood of pigs (Duroc × Landrace × Yorkshire) at 2-days post inoculation and before *Salmonella* infection. The analysis identified a total of 29 differentially expressed microRNAs, most of which are implicated in *Salmonella* infection and immunology signaling pathways. Joint analysis of the microRNA and mRNA transcriptomes identified 24 microRNAs with binding sites that were significantly enriched in 3′ UTR of differentially expressed mRNAs. Of these microRNAs, three were differentially expressed after *Salmonella* challenge in peripheral blood (ssc-miR-146a-5p, ssc-miR-125a, and ssc-miR-129a-5p). Expression of 23 targets of top-ranked microRNA, ssc-miR-146a-5p, was validated by real-time PCR. The effects of miR-146a, IFN-γ, and IL-6 on the regulation of fecal bacteria shedding counts in pigs were investigated by in vivo study with a *Salmonella* challenge model.

**Conclusions:**

The results indicated that induction of miR-146a in peripheral blood could significantly increase the fecal bacterial load, whereas IFN-γ had the reverse effect. These microRNAs can be used to identify targets for controlling porcine salmonellosis.

## Background

MicroRNAs are small, non-coding RNAs that regulate gene expression by target mRNAs at the post-transcriptional level (degradation or translational repression) [1, 2]. Differential expression of microRNAs has been implicated in a number of biological processes, such as cancer, development, growth [3, 4], and especially in the regulation of the host immune system [5, 6]. Subsequent studies suggested the roles of microRNAs in response to infection by pathogens [7, 8]. The *Salmonella enterica* serovar Typhimurium is a Gram-negative bacterium that causes gastroenteritis in both humans and animals.

In pigs, *Salmonella enterica* is the cause of asymptomatic carriage status to systemic febrile infection-related death varying with the ages of the animals [9]. *Salmonella* carrier pigs can transmit bacteria to the pig carcass in the slaughterhouse and contaminate the pork product, thus posing a significant threat to the human health [10]. Establishment of *Salmonella* carrying status is determined by the virulence of the bacteria as well as the genetic predisposition of the infected individual [11]. Several important *Salmonella* resistance microRNAs have been identified in animals, including miR-146 and miR-155 [12], let-7 [13], miR-29 [14], miR-128 [15], miR-15 [16], and more remaining to be discovered. These microRNAs are summarized in recent reviews [17–19]. Previous research on low versus persistent *Salmonella* shedding pigs at 2-days post inoculation (2 dpi) identified miR-214 and miR-331 as regulators of immune-related genes in peripheral blood [20] and thus demonstrated the important roles of microRNAs in buffering gene expression variation [21].

The miR-146a was found to be coordinately up regulated in immune cells in response to *Salmonella* infection [12]. It has been reported in mouse that the levels of miR-146a was significantly increased in T helper 1 cells and decreased in T helper 2 cells [22]. Further investigation showed that miR-146a was involved in lymphocytes cell fate determination [22]. The transcription of miR-146a might be regulated by the NF-κB and the STAT family [23]. Recent gene knock-out studies showed an increased percentage of INFγ-producing T-cell subset in the miR-146a-deficient mice [24]. Also, miR-146a was found to be inducible upon stimulation with lipopolysaccharide (LPS) in a NF-κB-dependent manner, and to target the TRAF6 and IRAK1, which encode downstream adapter molecules of Toll like receptors [25].

The scope of the study is to identify the differences in microRNA transcription abundance in peripheral blood samples collected before *Salmonella* Typhimurium inoculation (day 0) and early infection stage at 2 days post inoculation (2 dpi). A microRNA, miR-146a, was linked to porcine *Salmonella* shedding count which could help identify novel targets for controlling this human foodborne pathogen in pigs.

## Results

To identify porcine microRNAs that are differentially expressed in response to *Salmonella* Typhimurium challenge, microRNA transcriptomes were constructed for blood samples collected from three piglets (randomly selected from the nine pigs described in the Materials and Methods) at day 0 and 2 dpi. A total of 136,491,615 microRNA sequences were obtained. The six samples had an average of 22,748,602 reads per library; the number of reads ranged between 13,997,226 and 30,710,701 reads. The microRNA sequences were mapped to the 457 *Sus scrofa* microRNA mature sequences deposited in miRBase, and 185 microRNAs that were present across the six samples were identified. For transcriptome profile analysis of microRNA samples collected on day 0 and 2 dpi, a total of 29 differentially expressed microRNAs were identified based on the following criteria: 1) minimum read count of 20; 2) fold change ≥2.0, and 3) FDR ≤ 0.05 (Table 1). A total of 18 microRNAs were significantly downregulated, and five of these, namely, ssc-miR-16, ssc-miR-143-3p, ssc-miR-23b, and ssc-miR-744, were differentially expressed by at least four-fold. A total of 12 microRNAs were significantly upregulated, and five of these, namely, ssc-miR-155-5p ssc-miR-124a, ssc-miR-127, ssc-miR-26a, and ssc-miR-146a-5p, were differentially expressed by at least four-fold (Table 1).

**Table 1:** Differentially expressed microRNAs in response to *Salmonella* Typhimurium challenge

Accession No.	Name	Fold change	FDR	Sequence
MIMAT0007754	ssc-miR-16	−6.88	3.71E-05	UAGCAGCACGUAAAUAUUGGCG
MIMAT0022959	ssc-miR-155-5p	7.43	0.000113	UUAAUGCUAAUUGUGAUAGGGG
MIMAT0013879	ssc-miR-143-3p	−5.96	0.000194	UGAGAUGAAGCACUGUAGCUC
MIMAT0013893	ssc-miR-23b	−4.60	0.000214	AUCACAUUGCCAGGGAUUACCA
MIMAT0013932	ssc-miR-127	5.83	0.000322	UCGGAUCCGUCUGAGCUUGGCU
MIMAT0002135	ssc-miR-26a	5.20	0.000459	UUCAAGUAAUCCAGGAUAGGCU
MIMAT0002156	ssc-miR-124a	5.86	0.000571	UAAGGCACGCGGUGAAUGCCA
MIMAT0002157	ssc-miR-128	−3.98	0.000613	UCACAGUGAACCGGUCUCUUU
MIMAT0007755	ssc-miR-17-5p	−3.01	0.000617	CAAAGUGCUUACAGUGCAGGUAG
MIMAT0013865	ssc-let-7a	−3.90	0.000678	UGAGGUAGUAGGUUGUAUAGUU
MIMAT0002133	ssc-miR-23a	−3.57	0.001286	AUCACAUUGCCAGGGAUUUCC
MIMAT0013916	ssc-miR-34c	−3.58	0.001402	AGGCAGUGUAGUUAGCUGAUUGC
MIMAT0015708	ssc-miR-744	−4.32	0.00213	UGCGGGGCUAGGGCUAACAGCA
MIMAT0022963	ssc-miR-146a-5p	4.36	0.003514	UGAGAACUGAAUUCCAUGGGUU
MIMAT0013867	ssc-let-7 g	3.00	0.004261	UGAGGUAGUAGUUUGUACAGUU
MIMAT0007753	ssc-miR-15a	−3.22	0.004261	UAGCAGCACAUAAUGGUUUGU
MIMAT0013908	ssc-miR-92a	−3.44	0.004261	UAUUGCACUUGUCCCGGCCUGU
MIMAT0002152	ssc-let-7f-5p	−3.19	0.004365	UGAGGUAGUAGAUUGUAUAGUU
MIMAT0025361	ssc-miR-132	3.25	0.00443	UAACAGUCUACAGCCAUGGUCG
MIMAT0002167	ssc-miR-30c-5p	−3.36	0.00443	UGUAAACAUCCUACACUCUCAGC
MIMAT0002153	ssc-let-7i-5p	−2.88	0.0055	UGAGGUAGUAGUUUGUGCU
MIMAT0007757	ssc-miR-34a	−3.30	0.0055	UGGCAGUGUCUUAGCUGGUUGU
MIMAT0013875	ssc-miR-199a-3p	−3.10	0.008427	ACAGUAGUCUGCACAUUGGUUA
MIMAT0002165	ssc-miR-21-5p	2.71	0.010239	UAGCUUAUCAGACUGAUGUUGA
MIMAT0041605	ssc-miR-141	2.57	0.011832	UAACACUGUCUGGUAAAGAUG
MIMAT0025384	ssc-miR-874	−2.56	0.011895	CUGCCCUGGCCCGAGGGACCGAC
MIMAT0013897	ssc-miR-125a	2.65	0.014516	UCCCUGAGACCCUUUAACCUGUG
MIMAT0032108	ssc-miR-129a-5p	−2.71	0.025477	CUUUUUGCGGUCUGGGCUUGC
MIMAT0002155	ssc-miR-107	2.36	0.025666	AGCAGCAUUGUACAGGGCUAUCA
MIMAT0002151	ssc-let-7c	2.80	0.032272	UGAGGUAGUAGGUUGUAUGGUU

The microRNA profiling data were analyzed jointly with the mRNA transcriptome data to identify microRNAs whose binding sites were significantly enriched in the 3′ untranslated region (UTR) of differentially expressed mRNAs. A microRNA-regulator-(target gene) network was defined as a microRNA connected to a regulator, which is in turn connected to a set of target genes. These networks were created using experimentally validated binding sites deposited in the miRTarBase [26] and GREDB (http://www.thua45.cn/geredb/). The inputs are expression profiles of the target genes. The statistical problem was to test whether the microRNA binding sites in targets are enriched in the differentially expressed genes, and they can be formulated in a two-by-two contingency table. Significant enrichment values in the corresponding network and gene expression profile can be deduced using Fisher’s exact test. The mRNA transcriptome profiling data in porcine peripheral blood challenge with *Salmonella* bacteria was obtained from the NCBI GEO database with accession number GSE118150. Comparisons were made between basal and 2 days of *Salmonella* challenge. The input files were created with gene expression significantly changed in comparison with FDR ≤0.05 and marked as “diff”. The result indicated that the binding sites of 24 microRNAs were significantly enriched in the “diff” genes (Table 2). In these microRNAs, three were differentially expressed after *Salmonella* challenge in peripheral blood (ssc-miR-146a-5p, ssc-miR-125a, and ssc-miR-129a-5p, see Table 1 for details).

**Table 2:** Significantly enriched microRNAs in mRNA transcriptome of peripheral-blood challenged with *Salmonella*

Regulon	A^a^	B^b^	C^c^	D^d^	FDR
ssc-miR-146a-5p	187	620	1081	6808	1.89E-09
ssc-miR-125a	107	644	565	7029	4.11E-07
ssc-let-7e-5p	64	674	315	7204	0.000116
ssc-miR-466n-3p	85	665	502	7110	0.000195
ssc-miR-27a-3p	116	648	742	6983	0.000315
ssc-miR-3081-3p	24	698	78	7344	0.000381
ssc-miR-2139	24	698	77	7345	0.000403
ssc-miR-467 g	98	664	637	7057	0.001425
ssc-miR-669 m-3p	75	668	461	7125	0.001578
ssc-miR-466e-3p	94	665	608	7069	0.001627
ssc-miR-466d-3p	94	665	612	7067	0.001651
ssc-miR-298-5p	106	643	700	6890	0.001840
ssc-miR-297b-3p	94	665	608	7069	0.002827
ssc-miR-466a-3p	94	665	619	7065	0.002945
ssc-miR-466b-3p	55	679	322	7198	0.004303
ssc-miR-466c-3p	55	679	322	7198	0.004303
ssc-miR-466p-3p	55	679	322	7198	0.004303
ssc-miR-20a-5p	110	650	767	6971	0.006205
ssc-miR-5098	27	693	123	7321	0.006687
ssc-miR-181b-5p	109	636	769	6853	0.006835
ssc-miR-129a-5p	143	633	1071	6887	0.007193
ssc-miR-138-5p	48	679	265	7190	0.007297
ssc-miR-125b-5p	114	639	803	6865	0.007862
ssc-miR-19a-3p	83	649	554	6927	0.007940

^a^Number of microRNA targets in differential gene list

^b^Number of microRNA targets in non-differential gene list

^c^Number of non-microRNA targets in differential gene list

^d^Number of none-microRNA targets in none-differentially gene list

**Fig. 1:**
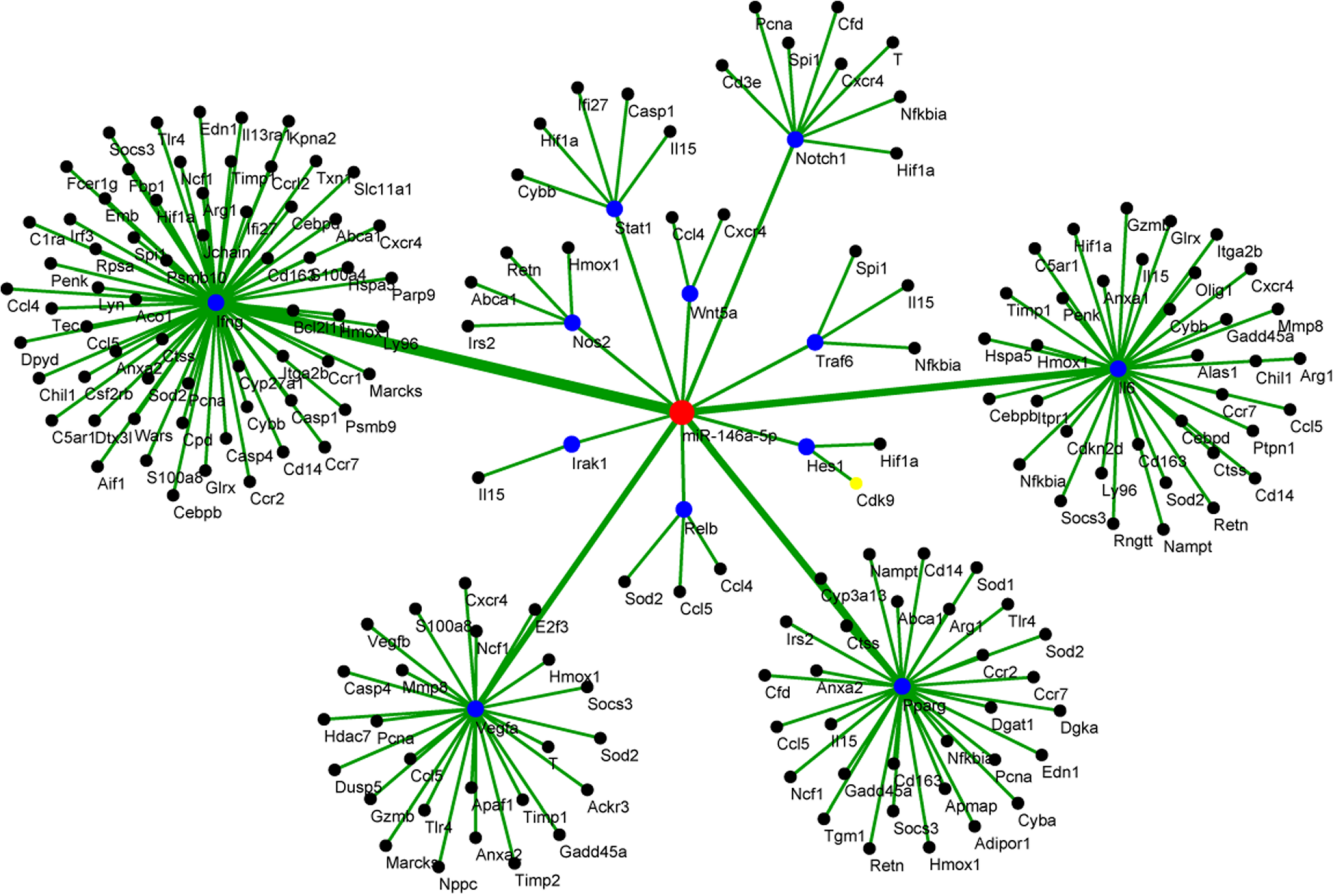
The miR-146a regulation network. The microRNA is plotted at the center of the graph (red). The gene regulators regulated by the microRNA are plotted around the microRNA (blue) with edges weighted by the number of target genes. The target genes are plotted as black nodes and labeled by the official gene symbol

There are a total of 807 miR-146a-5p target in the uploaded gene list (Ratio = 0.083) and a total of 187 miR-146a-5p target in the differentially expressed gene list (Ratio = 0.147) gives a highly significant *p* value of 1.89E-09 (Table 2). The top ranked microRNA, miR-146a-5p, can regulate 12 regulators, namely, INFG, IL6, VEGFA, PPARG, NOTCH1, STAT1, NOS2, TRAF6, RELB, WNT5A, HES1, and IRAK1. Among those regulators, INFG, IL6, VEGFA, and PPARG can regulate the largest number of target genes. A total of 64 IFNG targets, 36 IL6 targets, 25 VEGFA targets, and 31 PPARG targets were differentially expressed in peripheral blood after *Salmonella* challenge (Fig. 1).

**Fig. 2:**
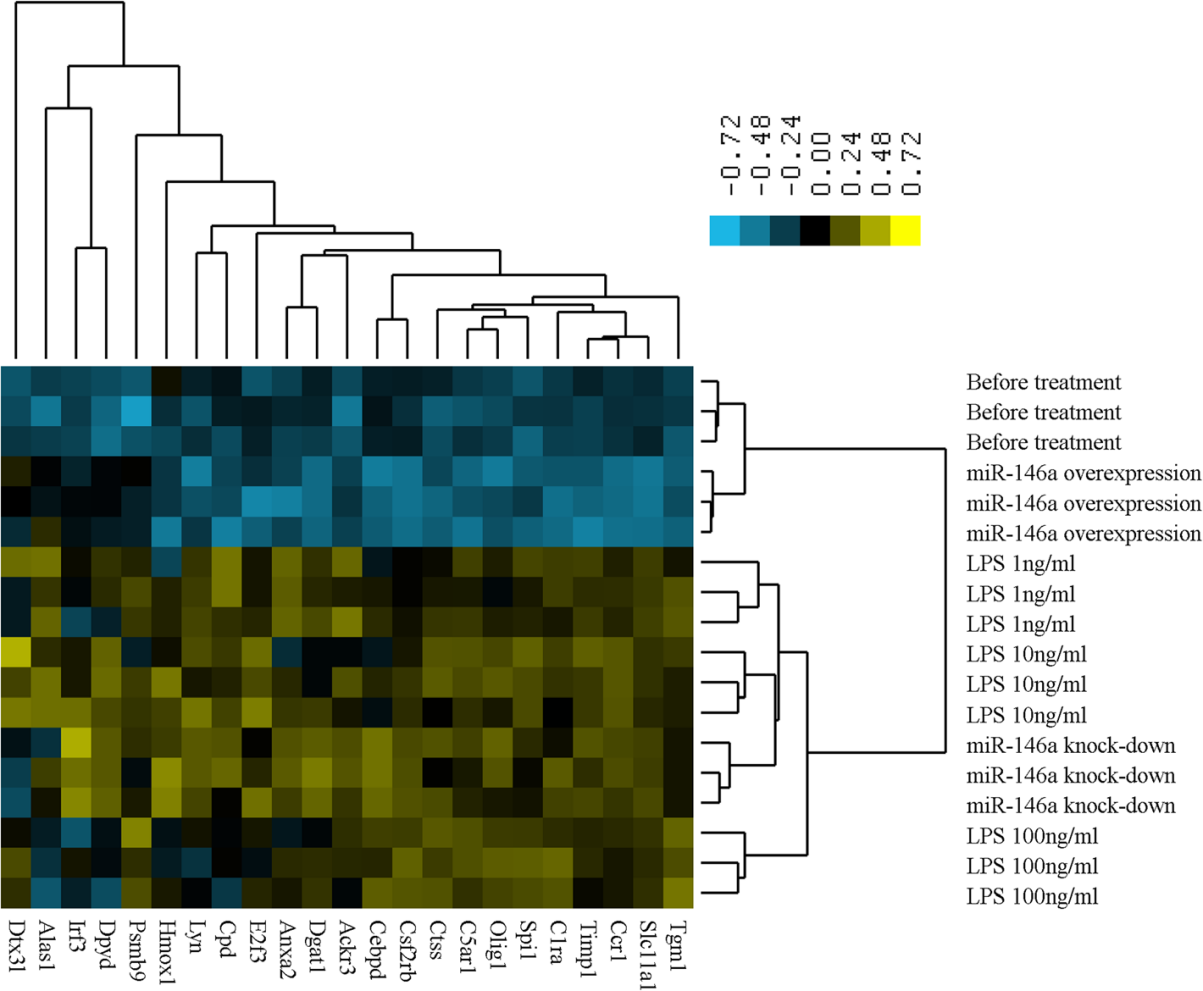
Hierarchical clustering of the gene expression data in PBMCs measured by real-time PCR. Cells were treated in vitro with three different doses of LPS (1 ng/ml, 10 ng/ml, and 100 ng/ml) and miR-146 overexpression or knockdown. Color codes of yellow, black, and blue represent expression levels of high, average, and low, respectively, across the treatments shown

To determine if the in vivo expression patterns of the 23 miR-146a target genes could be modeled by varying LPS levels, we performed real-time PCR after in vitro treatment in peripheral blood mononuclear cell (PBMCs) with three different doses of LPS (1 ng/ml, 10 ng/ml, and, 100 ng/ml), and miR-146a overexpression or knockdown. Samples were collected at 4 h post stimulation. A total of 21 genes were induced in response to LPS treatment, as RNA levels for these genes in the non-stimulated control were different from at least one dose of LPS stimulation. A total of 10 genes were down-regulated in response to overexpression with miR-146a, and 19 genes were up-regulated in response to knockdown with miR-146a. We used hierarchical clustering analysis to determine whether the LPS stimulation response pattern of the combined miR-146a target genes was similar to the patterns detected in miR-146a overexpression or knockdown, and if any similarity depended on the dosage of LPS used. Hierarchical clustering analysis of the average mRNA levels of the 23 miR-146a target genes indicated that the expression patterns of samples with miR-146a knockdown clustered with the LPS stimulation group (Fig. 2). When the LPS dose was increased, the expression pattern became less similar with miR-146a knockdown, and the most similar pattern seen upon LPS stimulation was at the 10 ng/ml dose. As expected, the expression pattern of miR-146a overexpression alone was unique and was neither similar to the LPS stimulation nor the miR-146 knockdown. The greatest similarity seen was in the samples before any treatment.

**Fig. 3:**
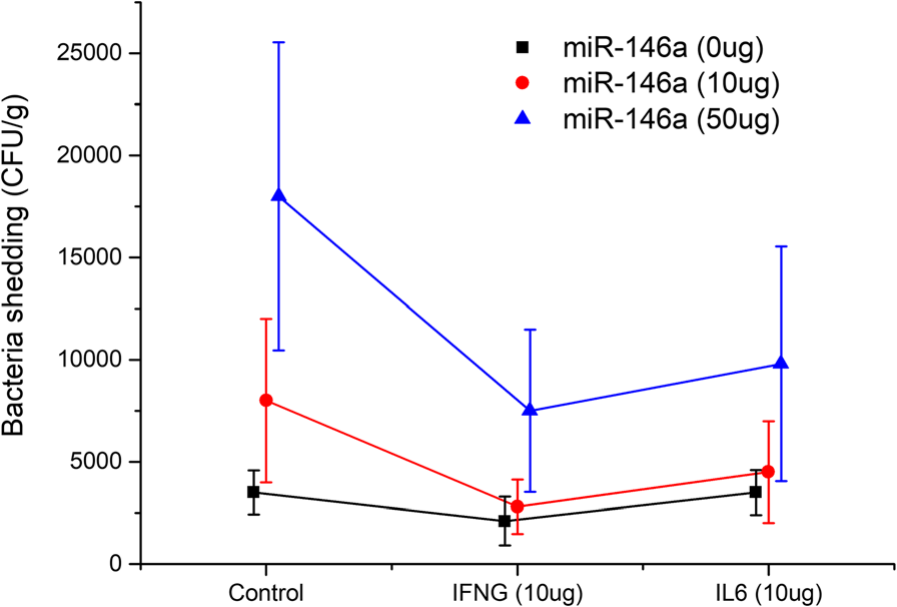
Effects of miR-146a, IFN-γ and IL-6 on *Salmonella* shedding counts in pigs. *Salmonella* shedding counts of a three (0 μg: black square, 10 μg: red cycle, and 50 μg: blue triangle) by three (Control, IFN-γ and IL-6) factor, completely randomized design with five animals in each group

To evaluate the potential effects of miR-146a in *Salmonella* infected pigs, measurements of *Salmonella* shedding counts were taken in *Salmonella* challenged pigs that had received either mock nanovector or miR-146a nanovector by jugular-vein injection (10 and 50 μg), twice in 1 day. Meanwhile, to show the involvement of the regulator gene, pigs were also treated with IFN-γ and IL-6 recombinant protein by jugular-vein injection (10 μg). The experiment was performed in a three (0, 10, and 50 μg) by three (Control, IFN-γ, and IL-6) factor completely randomized design with five *Salmonella*-free animals in each group. SAS GLM analysis showed an effect on *Salmonella* fecal shedding count for miR-146a and IFN-γ treatment (*p* < 0.06) but not for IL-6. A significant increase in *Salmonella* shedding counts was observed when pigs were treated with miR-146a nanovectors compared with the mock nanovector (Fig. 3). The shedding counts of the 50 μg miR-146a nanovector treatment group were 5.14 and 2.15 fold higher than the mock nanovector and 10 μg miR-146a nanovector treatment group, respectively (*p* < 0.05). There was no significant difference between the mock and 10 μg miR-146a nanovector treatment group. These results indicate that the promotive effects on shedding counts were caused by induction of the miR-146a nanovector. Notably, the *Salmonella* shedding count of IFN-γ treatment group was significantly lower than the control group (*p* < 0.05), whereas the IL-6 treatment group was not significantly different.

## Discussion

Several microRNAs have been reported to be differentially regulated following *Salmonella* infection. For example, miR-146 and miR-155 were demonstrated to be coordinately upregulated in immune cells [12]. Expression of the microRNA let-7 is downregulated in macrophage cells in response to *Salmonella* infection [13]. MiR-128 expression is upregulated in intestinal epithelial cells [15], while miR-15 is downregulated via inhibition by the transcription factor E2F1 [16]. In the presented study, both miR-146 and miR-155 were upregulated (4.36- and 7.43-fold, respectively) following *Salmonella* infection. On the other hand, let-7 s (let-7a, −g, −f, and -i) expression levels were downregulated, with fold changes ranging from 2.88 to − 3.9, and miR-15 was downregulated by 3.44-fold. The only exception is that, contrary to previously reported findings, miR-128 was downregulated by 3.9-fold. In our previous study, we showed that miR-143 expression was significantly downregulated in persistent shedding animals compared to low shedding animals after *Salmonella* infection. In addition, miR-26 expression was induced 10.5-fold in persistent shedding animals and was upregulated by 14-fold at 2 dpi [27]. Consistent with previous findings, our results showed that miR-143 expression was downregulated by 5.96-fold, while miR-26 expression was upregulated by 5.2-fold. Other microRNAs, such as miR-124, did not show evidence of direct involvement with *Salmonella* bacterial infection but have been predicted to play roles in targeting immune signaling pathways. MiR-124 expression is upregulated by 5.86-fold at 2 dpi. IQGAP2 was a predicted target of miR-124 and is downregulated by fivefold in persistent shedding pigs after *Salmonella* inoculation [28, 29]. IQGAP2 is associated with the CDC42 protein, which is known to regulate the MAPK signaling pathway. The observed upregulation of miR-124 expression and downregulation of IQGAP2 expression indicated that the CDC42-MAPK pathway is highly possibly inhibited after *Salmonella* infection. The microRNA responses to *Salmonella* challenge provide interesting hypotheses on the mechanisms underlying *Salmonella* infection in pigs.

The miR-146a was found to be a NF-κB-dependent gene and is responsible for regulated innate immune genes such as TRAF6 and IRAK1 [25]. Previous studies have shown that upregulation of miR-146a significantly inhibits LPS-induced IFN-γ expression in mouse splenic lymphocytes [30]. Also, miR-146a is upregulated during retinal pigment epithelium aging in mice and represses IL-6 expression in RPE cells [31]. IFN-γ was found to promote rapid acidification of phagolysosomes within infected macrophages and this low pH within the phagolysosome improves reactive nitrogen species production and leads to elimination of the pathogen [32]. The roles of IFN-γ in *Salmonella* infection have been reviewed by Gunjan et al. [33]. In brief, IFN-γ has been proved critical in mediating intestinal immunity to *Salmonella* typhimurium [34, 35]; IFN-γ also facilitated internalization and promoted early killing of *Salmonella* without employing oxidative burst [36]; furthermore, IFN-γ depleted intracellular iron levels in *Salmonella* typhimurium infected macrophages and exerted it’s anti-microbial effect [37]. In this study, the effects of miR-146a, IFN-γ, and, IL-6 on *Salmonella* fecal shedding counts in pig were investigated. Although the detailed mechanism of miR-146a regulation of IFN-γ and IL-6, and the establishment of different shedding status, remains unknown, it is clear that an appropriate innate immune response is required to defend an organism against *Salmonella* infection. However, if attenuated at post-transcription level, the response can be insufficient, causing the pathological manifestations of increased bacterial load and prolonged shedding status.

## Conclusions

The present study identified a total of 29 differentially expressed microRNAs responses in porcine peripheral blood after inoculation with the human foodborne pathogen *Salmonella enterica* serovar Typhimurium. Joint analysis of the microRNA and mRNA transcriptomes using binding sites enrichment analysis identified three microRNA candidates (ssc-miR-146a-5p, ssc-miR-125a, and ssc-miR-129a-5p) which were both differentially expressed after *Salmonella* challenge in peripheral blood and shown over-represented binding sites in differentially expressed mRNA list. In vivo study with a *Salmonella* challenge model indicated that induction of miR-146a in peripheral blood could significantly increase the fecal bacterial load by regulating IFN-γ targets.

## Methods

Nine crossbred (Duroc × Landrace × Yorkshire), conventionally raised, mixed gender piglets from three sows were weaned at 14 days of age and housed in an animal room under controlled and standardized conditions (animals were obtained from Da Bei Nong group, China). Animals were provided free access to standard food and water at 7 days before the experiment (average weight 6.83 ± 1.24kg, tested for *Salmonella* free twice). *Salmonella* Typhimurium cells (*Salmonella enterica* serovar Typhimurium strain LT2, ATCC 700720) were cultured in Luria broth and M9 minimal medium as previously described [27]. Piglets with *Salmonella*-negative fecal were challenged with *Salmonella* Typhimurium as previously described [28, 29]. Briefly, piglets were intranasally administered with 1 × 10^9^ colony forming units (CFUs) of *Salmonella* Typhimurium. Blood samples were obtained from the jugular vein of each animal before *Salmonella* inoculation (day 0) and 2 dpi following a previously described method [28, 29]. MicroRNA transcriptomes were sequenced for blood samples collected from three randomly selected piglets. After the experiment, the animals were combine treated with gentamicin and enrofloxacin for 7 days and tested for *Salmonella* free, then feeding in the isolation house for 30 days, and finally transferred to growing to finish farm.

Blood samples were immediately preserved in PAXgene blood RNA tubes containing the RNA stabilization reagent. Total RNA was isolated using the PAXgene Blood microRNA Kit according to the manufacturer’s instructions (Qiagen, USA). RNA quality was assessed using an Agilent 2100 Bioanalyzer (Agilent Technologies, USA), and RNA quantity was assessed using a Nanodrop spectrophotometer (Thermo Scientific, USA). Libraries were constructed using the Illumina TruSeq Small RNA Sample Prep Kit and were sequenced on an Illumina HiSeq2000 sequencer (50 bp and single-read) (Illumina Inc., USA) [38]. Raw data were submitted to the NCBI GEO database under the accession NO. GSE120266. Sequence reads with lengths ranging from 18 to 26 nucleotides were extracted. Identical sequences were counted, and microRNAs were identified by aligning the unique sequences with those of known mature microRNA sequences downloaded from miRBase release 22. Porcine sequences with no mismatches with known microRNAs were regarded as real porcine microRNAs and were assigned the same accession number as those in miRBase. Data normalization and differential microRNA expression analysis were conducted using the limma R package [39]. In brief, a design matrix was created which includes separate coefficients for control (day 0) and 2 dpi blood samples, and then extract the difference as a contrast. The differentially expressed miRNAs were controlled as false discovery rate (FDR) ≤ 0.05 and fold change ≥1.5 or ≤ 0.67.

The miR-146a was amplified from genomic DNA sequence using Pfu polymerase and inserted into the XhoI restriction endonuclease site in the pMSCV-puro expression vector (Clontech Laboratories) [40]. The sequence of the expression vector was confirmed by sequencing. The lipid-based nanovector for systemic miR-146a delivery was prepared using the method described previously [40]. A liposomal-based nanovector was chosen on the basis of the proved safety and efficacy of such constructions and the enhanced circulation stability in blood afforded by the presence of polyethylene glycol [41]. The recombinant porcine IFN-γ and IL-6 proteins were purchased from R&D systems (985-PI-050 and 686-PI-025). The in vivo study was performed in 45 crossbred (Duroc × Landrace × Yorkshire), conventionally raised, mixed-gender piglets at a population age of 21 days. The animals were inoculated with *Salmonella* and then given different kinds of treatment at the beginning of the experiment, and the *Salmonella* shedding count in feces was measured at 2 days post inoculation use Uthe’s method described previously [28]. The data were analyzed using SAS 9.4 GLM procedure.

## Data Availability

The datasets generated and analyzed during the current study are available in the GEO repository (GSE120266 and GSE118150).

## Abbreviations

CFU: Colony forming units
Dpi: Days post inoculation
FDR: False discovery rate
LPS: Lipopolysaccharide
PBMC: Peripheral blood mononuclear cell
UTR: Untranslated region
